# Use of Physiological Data From a Wearable Device to Identify SARS-CoV-2 Infection and Symptoms and Predict COVID-19 Diagnosis: Observational Study

**DOI:** 10.2196/26107

**Published:** 2021-02-22

**Authors:** Robert P Hirten, Matteo Danieletto, Lewis Tomalin, Katie Hyewon Choi, Micol Zweig, Eddye Golden, Sparshdeep Kaur, Drew Helmus, Anthony Biello, Renata Pyzik, Alexander Charney, Riccardo Miotto, Benjamin S Glicksberg, Matthew Levin, Ismail Nabeel, Judith Aberg, David Reich, Dennis Charney, Erwin P Bottinger, Laurie Keefer, Mayte Suarez-Farinas, Girish N Nadkarni, Zahi A Fayad

**Affiliations:** 1 The Dr Henry D Janowitz Division of Gastroenterology Icahn School of Medicine at Mount Sinai New York, NY United States; 2 Hasso Plattner Institute for Digital Health at Mount Sinai Icahn School of Medicine at Mount Sinai New York, NY United States; 3 Department of Genetics and Genomic Sciences Icahn School of Medicine at Mount Sinai New York, NY United States; 4 Center for Biostatistics Department of Population Health Science and Policy Icahn School of Medicine at Mount Sinai New York, NY United States; 5 The BioMedical Engineering and Imaging Institute Icahn School of Medicine at Mount Sinai New York, NY United States; 6 Department of Psychiatry Icahn School of Medicine at Mount Sinai New York, NY United States; 7 Pamela Sklar Division of Psychiatric Genomics Icahn School of Medicine at Mount Sinai New York, NY United States; 8 Department of Anesthesiology Perioperative and Pain Medicine Icahn School of Medicine at Mount Sinai New York, NY United States; 9 Department of Environmental Medicine and Public Health Icahn School of Medicine at Mount Sinai New York, NY United States; 10 Division of Infectious Diseases Icahn School of Medicine at Mount Sinai New York, NY United States; 11 Office of the Dean Icahn School of Medicine at Mount Sinai New York, NY United States; 12 Nash Family Department of Neuroscience Icahn School of Medicine at Mount Sinai New York, NY United States; 13 Department of Medicine Icahn School of Medicine at Mount Sinai New York, NY United States; 14 Charles Bronfman Institute for Personalized Medicine Icahn School of Medicine at Mount Sinai New York, NY United States; 15 Department of Diagnostic, Molecular and Interventional Radiology Icahn School of Medicine at Mount Sinai New York, NY United States

**Keywords:** wearable device, COVID-19, identification, prediction, heart rate variability, physiological, wearable, app, data, infectious disease, symptom, prediction, diagnosis, observational

## Abstract

**Background:**

Changes in autonomic nervous system function, characterized by heart rate variability (HRV), have been associated with infection and observed prior to its clinical identification.

**Objective:**

We performed an evaluation of HRV collected by a wearable device to identify and predict COVID-19 and its related symptoms.

**Methods:**

Health care workers in the Mount Sinai Health System were prospectively followed in an ongoing observational study using the custom Warrior Watch Study app, which was downloaded to their smartphones. Participants wore an Apple Watch for the duration of the study, measuring HRV throughout the follow-up period. Surveys assessing infection and symptom-related questions were obtained daily.

**Results:**

Using a mixed-effect cosinor model, the mean amplitude of the circadian pattern of the standard deviation of the interbeat interval of normal sinus beats (SDNN), an HRV metric, differed between subjects with and without COVID-19 (*P*=.006). The mean amplitude of this circadian pattern differed between individuals during the 7 days before and the 7 days after a COVID-19 diagnosis compared to this metric during uninfected time periods (*P*=.01). Significant changes in the mean and amplitude of the circadian pattern of the SDNN was observed between the first day of reporting a COVID-19–related symptom compared to all other symptom-free days (*P*=.01).

**Conclusions:**

Longitudinally collected HRV metrics from a commonly worn commercial wearable device (Apple Watch) can predict the diagnosis of COVID-19 and identify COVID-19–related symptoms. Prior to the diagnosis of COVID-19 by nasal swab polymerase chain reaction testing, significant changes in HRV were observed, demonstrating the predictive ability of this metric to identify COVID-19 infection.

## Introduction

COVID-19 has resulted in over 41 million infections and more than 1.1 million deaths [1]. The prolonged incubation period and variable symptomatology of SARS-CoV-2 have facilitated disease spread. Approximately 30%-45% of individuals infected with SARS-CoV-2 are asymptomatic and testing is generally being limited to symptomatic individuals [2-4]. Health care workers, characterized as any type of worker in a health care system, represent a vulnerable population, with a threefold increased risk of infection compared to the general population [5]. This increased risk of transmission is important in health care settings, where asymptomatic or presymptomatic health care workers can shed the virus, contributing to transmission within health care facilities and their own households [6].

Digital health technology offers an opportunity to address the limitations of traditional public health strategies aimed at curbing the spread of COVID-19 [7]. Smartphone apps are effective in using symptoms to identify people who may be infected with SARS-CoV-2; however, these apps rely on ongoing participant compliance and self-reported symptoms [8]. Wearable devices are commonly used for remote sensing, and they provide a means to objectively quantify physiological parameters, including heart rate, sleep, activity, and measures of autonomic nervous system (ANS) function (eg, heart rate variability [HRV]) [9]. The addition of physiological data from wearable devices to symptom-tracking apps has been shown to increase the ability to identify people infected with SARS-CoV-2 [10].

HRV is a physiological metric that provides insight into the interplay between the parasympathetic and sympathetic nervous systems that modulate cardiac contractility and cause variability in the beat-to-beat intervals [11]. HRV exhibits a 24-hour circadian pattern, with relative sympathetic tone during the day and parasympathetic activity at night [12-14]. Changes in this circadian pattern can be leveraged to identify different physiological states. Several studies have demonstrated that lower HRV, indicating increased sympathetic balance, is a reliable predictor of infection onset [15,16]. However, HRV and its dynamic changes over time have not been evaluated as a marker or predictor of COVID-19. In response to the COVID-19 pandemic, we launched the Warrior Watch Study, employing a novel smartphone app to remotely enroll and monitor health care workers throughout the Mount Sinai Health System in New York City, a site of initial case surge. This digital platform enables the delivery of remote surveys to Apple iPhones and passive collection of Apple Watch data, including HRV. The aim of this study is to determine if SARS-CoV-2 infections can be identified and predicted prior to a positive test result using the longitudinal changes in HRV metrics derived from individuals’ Apple Watch data.

## Methods

### Study Design

The primary aim of the study was to determine whether changes in HRV can differentiate participants who are infected and not infected with SARS-CoV-2. The secondary aim was to observe if changes in HRV can predict the development of SARS-CoV-2 infection prior to diagnosis by a SARS-CoV-2 nasal swab polymerase chain reaction (PCR) test. The exploratory aims were to (1) determine whether changes in HRV can identify the presence of COVID-19–related symptoms; (2) determine whether changes in HRV can predict the development of COVID-19–related symptoms; and (3) evaluate how HRV changed throughout the infection and symptom period.

Health care workers in the Mount Sinai Health System were enrolled in an ongoing prospective observational cohort study. Eligible participants were aged ≥18 years, were current employees in the Mount Sinai Health System, had an iPhone Series 6 or higher, and had or were willing to wear an Apple Watch Series 4 or higher. Participants were excluded if they had an underlying autoimmune disease or were taking medications known to interfere with ANS function. A positive COVID-19 diagnosis was defined as a positive SARS-CoV-2 nasal swab PCR test reported by the participant. Daily symptoms were collected, including fever and chills, feeling tired or weak, body aches, dry cough, sneezing, runny nose, diarrhea, sore throat, headache, shortness of breath, loss of smell or taste, itchy eyes, none, or other. This study was approved by the Institutional Review Board at The Icahn School of Medicine at Mount Sinai.

### Study Procedures

Participants downloaded the custom Warrior Watch app to complete eligibility questionnaires and sign an electronic consent form. Participants completed an app-based baseline assessment collecting demographic information, prior COVID-19 diagnosis history, occupation, and medical history and were then followed prospectively through the app. Daily survey questionnaires captured COVID-19–related symptoms, symptom severity, SARS-CoV-2 nasal swab PCR test results, serum SARS-CoV-2 antibody test results, and daily patient care–related exposure (Table S1 in Multimedia Appendix 1). Participants performed their normal activities throughout the study and were instructed to wear the Apple Watch for a minimum duration of 8 hours per day.

### Wearable Monitoring Device and Autonomic Nervous System Assessment

HRV was measured via the Apple Watch Series 4 or 5, which are commercially available wearable devices. Participants wore the device on the wrist and connected it via Bluetooth to their iPhone. The Apple Watch is equipped with an enhanced photoplethysmogram optical heart sensor that combines a green light-emitting diode paired with a light-sensitive photodiode generating time series peaks that correlate with the magnitude of change in the green light generated from each heartbeat [17]. Data are filtered for ectopic beats and artifacts. The time difference between heartbeats is classified as the interbeat interval (IBI), from which HRV is calculated. The Apple Watch and the Apple Health app automatically calculate HRV using the standard deviation of the IBI of normal sinus beats (SDNN), measured in milliseconds. This time domain index reflects both sympathetic and parasympathetic nervous system activity and is calculated by the Apple Watch during ultra–short-term recording periods of approximately 60 seconds [11]. The Apple Watch generates several HRV measurements throughout a 24-hour period. HRV metrics are stored in a locally encrypted database accessible through the iPhone Health app, which is retrieved through our custom Warrior Watch app. Data are transferred from the iPhone and Apple Watch upon completion of the e-consent form and any survey in the app. Wearable data are stored locally, enabling retrieval on days when the surveys are not completed by the participants.

### Statistical Analysis

#### HRV Modeling

The HRV data collected through the Apple Watch were characterized by a circadian pattern, a sparse sampling over a 24-hour period, and nonuniform timing across days and participants. These characteristics bias easily derived features, including mean, maximum, and minimum, creating the need to derive methods that model the circadian rhythm of HRV. A cosinor model was used to model the daily circadian rhythm over a 24-hour period with the following nonlinear function:

Y(t) = M + *Acos*(2*π*t/τ + *ϕ*) + e_i_(t) **(1)**

where τ is the period (τ=24 h); M is the midline statistic of rhythm (MESOR), a rhythm-adjusted mean; A is the amplitude, a measure of half of the extent of variation within a day; and Φ is the acrophase, a measure of the time at which overall high values recur on each day (Figure S1 in Multimedia Appendix 1). This nonlinear model with three parameters has the advantage of being easily transformed into a linear model by recoding the time (t) in two new variables x and z as *x* = sin(2*π*t/*τ*), *z* = sin(2*π*t/*τ*). HRV can then be written as follows:

Y(t) = M + *β*x_t_ + *γ*z_t_ + e_i_(t) **(2)**

where the linear coefficients *β*, *γ* of the linear model in Equation 2 are related to the nonlinear parameters of the nonlinear model in Equation 1 by *β = Asin*(*ϕ*) and *γ =* –*Asin*(*ϕ*). One can estimate the linear parameters *β*, *γ* and then obtain A and *ϕ* as


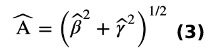



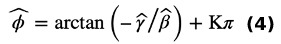


We took advantage of the longitudinal structure of the data to identify a participant-specific daily pattern, and we then measured departures from this pattern as a function of COVID-19 diagnosis or other relevant covariates. To do this, we used a mixed-effect cosinor model, where the HRV measure of participant *i* at time *t* can be written as follows:

HRV_it_ = (M + *β*.x_it_+*γ.*z_it_)+*W*_it_.*θ*_i_+e_i_(t), e_i_(t)~N(0,s) **(5)**

where M, *β*, and *γ* are the population parameters (fixed effects) and *θ*_i_ is a vector of random effects that is assumed to follow a multivariate normal distribution *θ*_i_~N(0,Σ). In this context, the introduction of random effects intrinsically models the correlation due to the longitudinal sampling. To measure the impact of any covariate C on the participants’ daily curve, we can introduce these covariates as fixed effects, as their interactions with x and z:

HRV_it_ = M + *a*_O_C_i_ + (*β* + *a*_2_C_i_).x_it_ + (*γ* + *a*_3_C_i_).z_it_ + *W_it_*.*θ*_i_ + e_i_(t) **(6)**

The model parameters and the standard errors of Equation 6 can be estimated via maximum likelihood or reweighted least squares (REWL), and hypothesis testing can be conducted for any comparison that can be written as a linear function of the *a*, *β*, and *γ* parameters.

However, to test if the cosinor curve, defined by the nonlinear parameters M, A, and *Φ* in Equation 1, differs between the populations defined by the covariate C, we proposed a bootstrapping procedure in which for each resampling iteration, we (1) fit a linear mixed-effect model using REWL; (2) estimated the marginal means by obtaining the linear parameters for each group defined by covariate C; (3) used the inverse relationship to estimate marginal means M, A, and *Φ* for each group defined by C; and (4) defined the bootstrapping statistics as the pairwise differences of M, A, and *Φ* between groups defined by C. For these iterations, the confidence intervals for the nonlinear parameter were defined using standard bootstrap techniques, and the *P* values derived for the differences in each nonlinear parameter between groups were defined by C_i_. Age and sex were included as covariates in the HRV analyses and admitted as invariant and time-variant covariates.

#### Association and Prediction of COVID-19 Diagnosis and Symptoms

The relationship between a COVID-19 diagnosis and the change in the HRV curves was evaluated. To test this association, we defined the time variant covariate C_it_ for participant i at time t as follows:


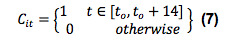


HRV metrics for the 14 days following the time of the first positive SARS-CoV-2 nasal swab PCR test were used to define the positive SARS-CoV-2 infection window. To evaluate the predictive ability of changes in HRV prior to a COVID-19 diagnosis and to explore its changes during the infection period, the time variant covariate was used to characterize the following 4 groups: healthy uninfected individuals (t<t_0_ – 7), 7 days before COVID-19 diagnosis (t≥t_0_ – 7, t<t_0_), the first 7 days post–COVID-19 diagnosis (t_0_≤t<t_0_ + 7), and 7-14 days postdiagnosis (t_0_ + 7≤t<t_0_ + 14).

To determine the association between COVID-19 symptoms and changes in HRV metrics, we defined being symptomatic as the first day of a reported symptom and compared this to all other days. To evaluate the predictive ability of HRV to identify upcoming symptom days and to explore its changes over time, the time variant covariate was used to characterize the following 4 groups: healthy asymptomatic individuals for t<t_0_ – 1, 1 day before COVID-19 symptoms (t≥t_0_ – 1, t<t_0_), the first day of COVID-19 symptoms (t_0_≤t<t_0_ + 1) and 1 day post–COVID-19 symptom development (t_0_ + 1≤t<t_0_ + 2).

## Results

### Participant Demographics

We enrolled 297 participants between April 29 and September 29, 2020, when the data were censored for analysis (Table 1). The median age at enrollment was 36 years (SD 9.8), and 69.4% of participants (204/297) were women. Of the 297 participants, 20 (6.7%) reported having a positive SARS-CoV-2 nasal swab PCR test prior to enrollment, while 28 participants (9.4%) reported having a positive blood antibody test prior to joining the study. The median duration of follow-up was 42 days (range 0-152 days). A median of 28 HRV samples (range 1-129) were obtained per participant. Study compliance over the follow-up period, defined as participants answering over 50% of daily surveys, was 70.4%.

**Table 1:** Baseline demographics of the study participants at enrollment (N=297).

Characteristic		Value
	Age (years), mean (SD)	36.3 (9.8)
	BMI (kg/m^2^), mean (SD)	25.6 (5.7)
	Female gender, n (%)	204 (69.4)
**Race, n (%)**		
	Asian	73 (24.6)
	Black	29 (9.8)
	White	108 (36.4)
	Other	43 (14.5)
Hispanic ethnicity, n (%)		44 (14.8)
Baseline positive SARS-CoV-2 nasal swab PCR^a^ test, n (%)		20 (6.7)
Baseline positive SARS-CoV-2 serum antibody test, n (%)		28 (9.4)
**Occupation^b^, n (%)**		
	Clinical nontrainee	198 (68.0)
	Clinical trainee	36 (12.4)
	Nonclinical staff	57 (19.6)
**Baseline smoking status, n (%)**		
	Current or past smoker	35 (11.9)
	Nonsmoker or rare smoker	259 (88.1)
Baseline immune suppressing medication, n (%)		4 (1.4)

^a^PCR: polymerase chain reaction.

^b^Clinical trainee is defined as a resident or fellow; clinical nontrainee is defined as a health care worker reporting at least one patient-facing day during follow-up, exclusive of residents and fellows; nonclinical staff is defined as a health care worker who did not report a patient-facing day during follow-up.

### Identification and Prediction of COVID-19 Diagnosis

**Figure 1:**
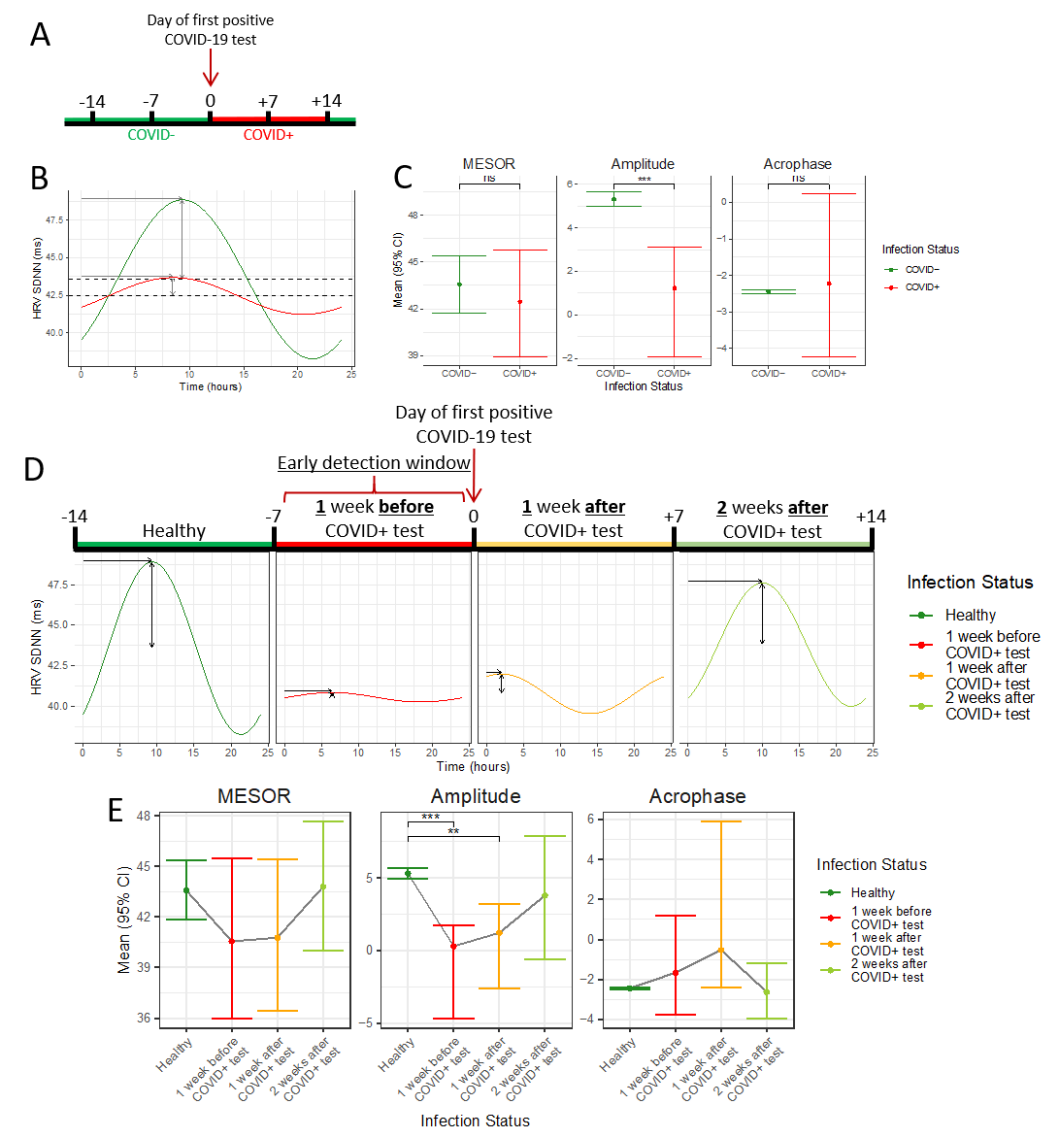
Relationship between HRV circadian rhythm and COVID-19 status. Timeline (A) illustrates HRV measures from the time of COVID-19 diagnosis via nasal swab PCR test and during the following 2 weeks after subjects were deemed to be COVID-19–positive and were compared with measurements outside this window, where subjects were deemed to be COVID-19–negative. Daily HRV rhythm (B) on days with positive and negative COVID-19 diagnoses. Plots (C) showing the means and 95% confidence intervals for the parameters defining the circadian rhythm, acrophase, amplitude and MESOR, on days with positive and negative COVID-19 diagnoses. Daily HRV patterns (D, E) for days on which subjects were healthy, 7 days before a positive COVID-19 test, 7 days after a positive COVID-19 test, and 7-14 days after a positive COVID-19 test. Means and 95% confidence intervals for the acrophase, amplitude, and MESOR of the HRV measured on days when participants were healthy, 7 days before a positive COVID-19 test, 7 days after a positive COVID-19 test, and 7-14 days after a positive COVID-19 test. *P<.10, **P<.05, ***P<.01, ****P<.001, ns: not significant. HRV: heart rate variability; MESOR: midline statistic of rhythm; SDNN: standard deviation of the interbeat interval of normal sinus beats.

Participants classified as not having a COVID-19 diagnosis during follow-up included those with and without a diagnosis of COVID-19 prior to study enrollment. There was no significant difference in the mean MESOR, acrophase, or amplitude of the circadian SDNN pattern of participants with a positive nasal swab PCR test prior to enrollment compared to those who were never diagnosed with COVID-19. This supports the inclusion of participants with a prior COVID-19 diagnosis in our analysis (Table S2 in Multimedia Appendix 1). During the follow-up period, 13/297 participants (4.4%) reported a positive SARS-CoV-2 nasal swab PCR test, with the date of diagnosis corresponding with the reported date of the positive test. The mean MESOR, acrophase, and amplitude of the circadian SDNN pattern in participants who were and were not diagnosed with COVID-19 during follow-up are described in Table 2. A significant difference in the circadian pattern of SDNN was observed in participants diagnosed with COVID-19 compared to those without a COVID-19 diagnosis. There was a significant difference (*P*=.006) between the mean amplitude of the circadian pattern of SDNN in participants with COVID-19 (1.23 milliseconds, 95% CI –1.94 to 3.11) and without COVID-19 (5.30 milliseconds, 95% CI 4.97 to 5.65). No difference was observed between the MESOR (*P*=.46) or acrophase (*P*=.80) in these two infection states (Figure 1A-C). Similar findings were observed when this analysis was repeated to include only participants who had either a positive (13/297, 4.4%) or negative (108/297, 36.4%) SARS-CoV-2 nasal swab PCR test during the follow-up period, excluding participants who reported never being tested (Table S3 in Multimedia Appendix 1).

The mean MESOR, acrophase, and amplitude of the circadian SDNN pattern for those without COVID-19, those during the 7 days prior to a COVID-19 diagnosis, participants during the 7 days after a COVID-19 diagnosis, and those during the 7-14 days after a COVID-19 diagnosis are described in Table 3. Significant changes in the circadian SDNN pattern were observed in participants during the 7 days prior to and the 7 days after a diagnosis of COVID-19 when compared to uninfected participants. There was a significant difference between the amplitude of the SDNN circadian rhythm between uninfected participants (5.31 milliseconds, 95% CI 4.95 to 5.67) compared to individuals during the 7-day period prior to a COVID-19 diagnosis (0.29 milliseconds, 95% CI –4.68 to 1.73; *P*=.01) and participants during the 7 days after a COVID-19 diagnosis (1.22 milliseconds, 95% CI –2.60 to 3.25; *P*=.01). There were no other significant differences between the MESOR, amplitude, and acrophase of the circadian rhythm of the SDNN observed between healthy individuals, individuals 7 days before a COVID-19 diagnosis, individuals 7 days after a COVID-19 diagnosis, and individuals 7-14 days after infection (Figure 1D-E).

Of the 13 subjects diagnosed with COVID-19 during follow up, 6 reported symptoms at some point during the study period. Only 4 subjects had symptomatic COVID-19 infections, reporting symptoms between 7 days prior and 14 days after a positive SARS-CoV-2 nasal swab PCR test. Comparing participants with and without symptomatic COVID-19, no significant differences between the MESOR (28.58 milliseconds, 95% CI 18.61 to 38.56; 37.71 milliseconds, 95% CI 30.65 to 44.98, *P*=.11), amplitude (1.15 milliseconds, 95% CI –2.63 to 3.21; 1.68 milliseconds, 95% CI –1.13 to 3.95, *P*=.76) and acrophase (–1.92 milliseconds, 95% CI –3.68 to –0.02; –2.49 milliseconds, 95% CI –4.37 to –0.33, *P*=.62) of the circadian rhythm of SDNN were observed, respectively.

**Table 2:** HRV parameters in participants with and without COVID-19 diagnoses based on SARS-CoV-2 nasal swab PCR tests.

Parameter	Parameter (milliseconds), mean (95% CI)			Difference (95% CI)		*P* value
	Participants not diagnosed with COVID-19	Participants diagnosed with COVID-19				
MESOR^a^	43.57 (41.40 to 45.40)	42.46 (38.90 to 45.79)	–1.12 (–4.22 to 1.73)		.46	
Amplitude	5.30 (4.97 to 5.65)	1.23 (–1.94 to 3.11)	–4.07 (–7.29 to –2.07)		*.006* ^b^	
Acrophase	–2.44 (–2.49 to –2.39)	–2.23 (–2.22 to –4.24)	0.22 (–1.74 to 2.43)		.80	

^a^MESOR: midline statistic of rhythm.

^b^Italic text indicates statistical significance.

**Table 3:** Comparison of heart rate variability parameters based on the time periods before and after diagnosis.

Parameter and first period relative to COVID-19 diagnosis		Value (milliseconds), mean (95% CI)	Second period relative to COVID-19 diagnosis	Value (milliseconds), mean (95% CI)	Difference (95% CI)	*P* value
**MESOR^a^**						
	7 days before	40.56 (35.98 to 45.46)	Uninfected	43.58 (41.88 to 45.37)	–3.03 (–6.98 to 1.02)	.13
	7 days after	40.77 (36.44 to 45.42)	Uninfected	43.58 (41.88 to 45.37)	–2.81 (–6.73 to 1.10)	.17
	7-14 days after	43.80 (40.01 to 47.65)	Uninfected	43.58 (41.88 to 45.37)	0.22 (–3.39 to 3.73)	.89
	7 days before	40.56 (35.98 to 45.46)	7-14 days after	43.80 (40.01 to 47.65)	–3.24 (–9.63 to 3.33)	.32
	7 days after	40.77 (36.44 to 45.42)	7-14 days after	43.80 (40.01 to 47.65)	–3.03 (–6.98 to 1.02)	.13
	7 days after	40.77 (36.44 to 45.42)	7 days before	40.56 (35.98 to 45.46)	0.217 to (–3.39 to 3.73)	.89
**Amplitude**						
	7 days before	0.29 (–4.68 to 1.73)	Uninfected	5.31 (4.95 to 5.67)	–5.02 (–10.14 to –3.58)	*.01* ^b^
	7 days after	1.22 (–2.60 to 3.25)	Uninfected	5.31 (4.95 to 5.67)	–4.09 (–7.87 to –1.93)	*.01*
	7-14 days after	3.80 (–0.64 to 7.88)	Uninfected	5.31 (4.95 to 5.67)	–1.51 (–5.79 to 2.35)	.48
	7 days before	0.29 (–4.68 to 1.73)	7-14 days after	3.80 (–0.64 to 7.88)	–3.51 (–10.50 to 0.22)	.20
	7 days after	1.22 (–2.60 to 3.25)	7-14 days after	3.80 (–0.64 to 7.88)	–2.58 (–8.44 to 2.08)	.34
	7 days after	1.22 (–2.60 to 3.25)	7 days before	0.29 (–4.68 to 1.73)	0.93 (–1.92 to 5.83)	.58
**Acrophase**						
	7 days before	–1.67 (–3.78 to 1.19)	Uninfected	–2.44 (–2.49 to –2.39)	0.78 (–1.4 to 3.62)	.45
	7 days after	–0.53 (-2.39-5.89)	Uninfected	–2.44 (–2.49 to –2.39)	1.92 (0.03 to 8.13)	.48
	7-14 days after	–2.63 (–3.95 to 1.19)	Uninfected	–2.44 (–2.49 to –2.39)	–0.19 (–1.39 to 1.16)	.70
	7 days before	–1.67 (–3.78 to 1.19)	7-14 days after	–2.63 (–3.95 to 1.19)	0.96 (–1.85 to 4.32)	.55
	7 days after	–0.53 (–2.39 to 5.89)	7-14 days after	–2.63 (–3.95 to 1.19)	2.10 (0.10 to 8.29)	.35
	7 days after	–0.53 (–2.39 to 5.89)	7 days before	–1.67 (–3.78 to 1.19)	1.14 (–1.34 to 7.27)	.58

^a^MESOR: midline statistic of rhythm.

^b^Italic text indicates statistical significance.

### Identification and Prediction of COVID-19 Symptoms

**Figure 2:**
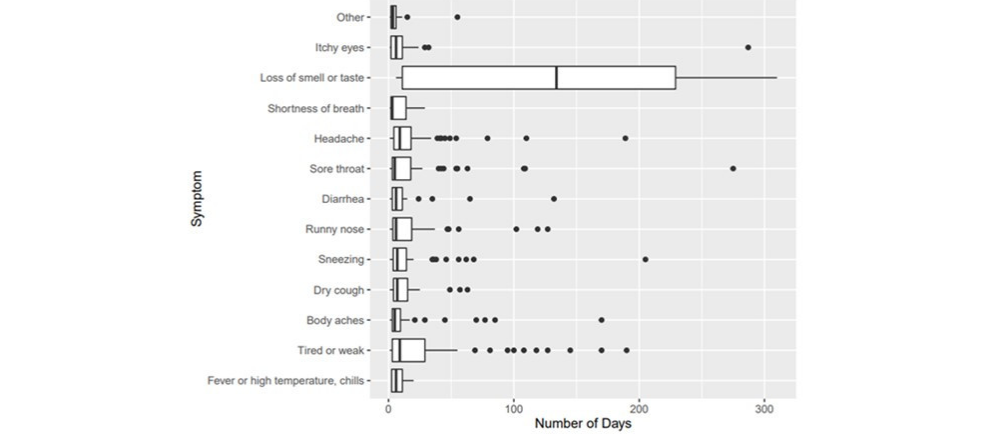
Number of symptom days per participant when evaluating days on which participants reported symptoms.

Of the 297 participants, 165 (55.6%) reported developing a symptom during the follow-up period, with the greatest number of participants reporting feeling tired or weak (n=87, 29.3%), followed by headaches (n=82, 27.6%) and sore throat (n=60, 20.2%) (Table 4). Evaluating the days on which participants experienced symptoms, we found that loss of smell or taste was reported the most frequently, with a mean of 138 days. This was followed by feeling tired or weak, reported for a mean of 25 days, and runny nose, reported for a mean of 19.5 days (Figure 2).

**Figure 3:**
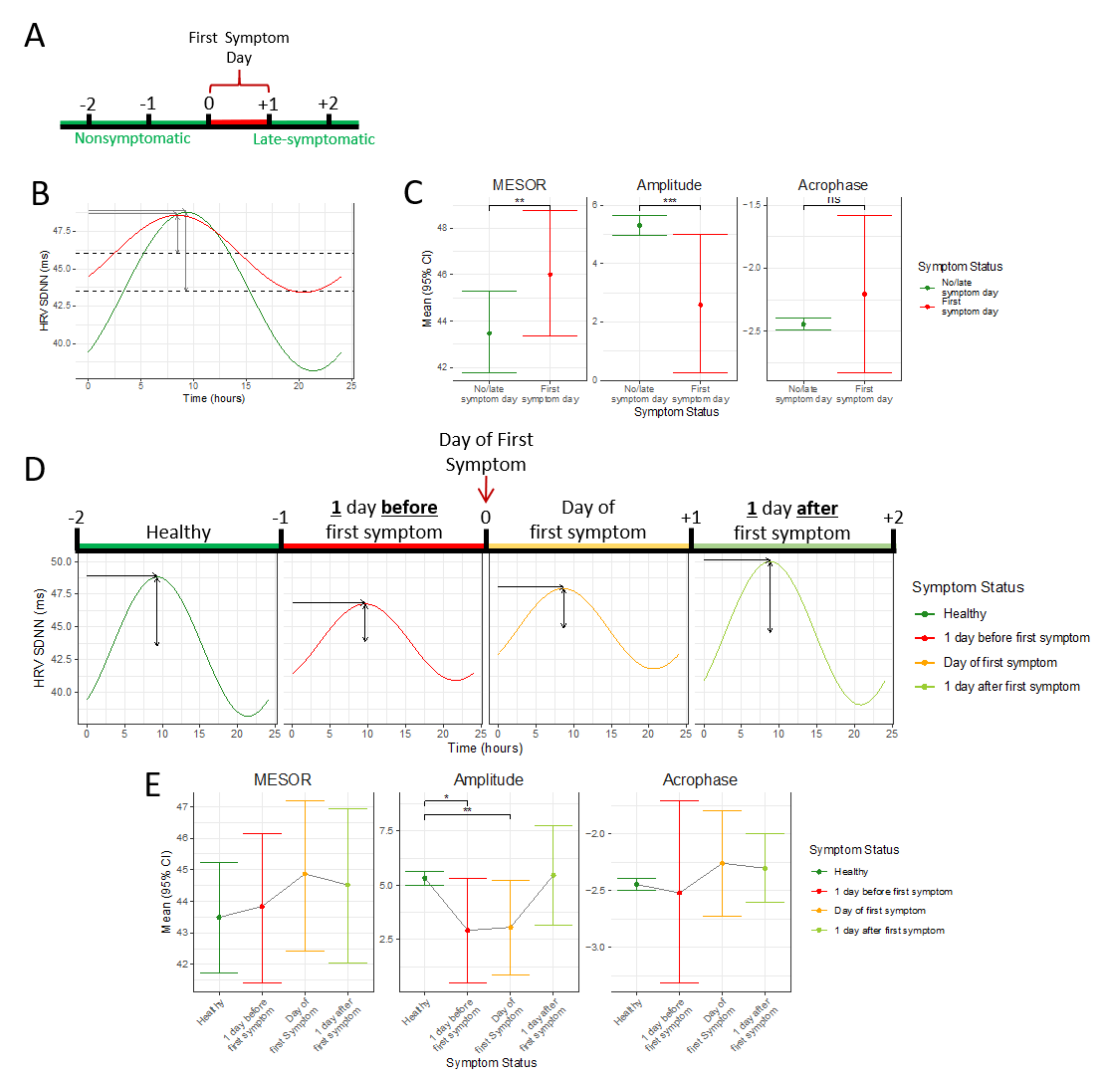
Relationship between HRV circadian rhythm and symptom onset. Timeline (A) illustrates the timing of symptom onset; the HRV profiles of the day of the first symptom are compared to all other days. Daily HRV rhythm (B) on the day of the first symptom and nonsymptomatic or late-symptom days. Plots (C) showing means and 95% confidence intervals for the parameters defining the circadian rhythm, acrophase, amplitude, and MESOR, on first symptom and nonsymptomatic or late-symptomatic days. Daily HRV pattern (D) for nonsymptomatic or late-symptomatic days, the day before the first symptom, the day of the first symptom, and the day after the first symptom. Means and 95% confidence intervals for the acrophase, amplitude, and MESOR of the HRV measured on nonsymptomatic or late-symptomatic days, the day before the first symptom, the day of the first symptom, and the day after the first symptom. *P<.10, **P<.05, ***P<.01, ****P<.001, ns, not significant. HRV: heart rate variability; MESOR: midline statistic of rhythm; SDNN: standard deviation of the interbeat interval of normal sinus beats.

The mean MESOR, acrophase, and amplitude observed in the circadian SDNN patterns of participants on the first day they experienced a symptom and on all other days of follow-up are reported in Table 5. There was a significant difference in the circadian SDNN pattern between participants on the first day a symptom was reported compared to all other days of follow-up. Specifically, there was a significant difference (*P*=.01) between the mean MESOR of the circadian SDNN pattern on the first day of symptoms (46.01 milliseconds, 95% CI 43.37 to 48.77) compared to all other days (43.48 milliseconds, 95% CI 41.77 to 45.27). Similarly, there was a significant difference (*P*=.01) between the mean amplitude of the circadian SDNN pattern on the first day of symptoms (2.58 milliseconds, 95% CI 0.26-5.00) compared to all other days (5.30 milliseconds, 95% CI 4.95-5.66) (Figure 3A-C). Out of the 165 participants reporting symptoms during the follow-up period, 36 participants (21.2%) reported experiencing a symptom graded as 6 or higher on a 10-point scale. The impact of the severity of a symptom on the HRV was evaluated by comparing HRV metrics in participants with symptoms graded as a 6 or higher versus those with symptoms graded 5 or less. There was a significant difference between the mean amplitude (*P*=.02) and MESOR (*P*=.01) of the circadian SDNN pattern in subjects with low symptom severity (amplitude 9.39 milliseconds, 95% CI 7.41 to 11.02; MESOR 42.82 milliseconds, 95% CI 38.65 to 47.19) and high symptom severity (amplitude 4.74 milliseconds, 95% CI 0.75 to 8.40; MESOR 36.15 milliseconds, 95% CI 29.11 to 43.34). There was no significant difference (*P*=.84) in the acrophase between those with low symptom severity (–2.43 ms, 95% CI –2.59 to –2.26) and high symptom severity (–2.49 milliseconds, 95% CI –3.19 to –1.77).

The mean MESOR, acrophase, and amplitude observed in the circadian SDNN patterns of participants on the day before symptoms developed, on the first day of the symptom, on the day following the first day of the symptom, and on all other days are reported in Table 6. Significant changes in the circadian SDNN pattern were observed, specifically in the mean amplitude (*P*=.04), when comparing participants on the first day of the symptom (3.07 milliseconds, 95% CI 0.88 to 5.22) to all other days (5.32 milliseconds, 95% CI 4.99 to 5.66). Excluded from this analysis were the day prior to and the day after the first symptomatic day. Changes in SDNN characteristics trended toward significance prior to the development of symptoms. Specifically, the differences in the mean amplitude of the circadian SDNN pattern trended toward significance when comparing the day prior to symptom development (2.92 milliseconds, 95% CI 0.50 to 5.33) with all other days (5.32 milliseconds, 95% CI 4.99 to 5.66; *P*=.06). Again, the first day of the symptom and the day after the first symptomatic day were excluded from the analysis. Additionally, there was a trend toward significance when comparing the amplitude of the SDNN circadian pattern between participants during the first day of the symptom (3.07 milliseconds, 95% CI 0.88 to 5.22) with that one day after the first symptom was reported (5.47 milliseconds, 95% CI 3.16 to 7.76; *P*=.56). Excluded from the analysis were the day prior to symptom development and all other days. There were no other significant differences between the MESOR, amplitude, or acrophase of the circadian rhythm of the SDNNs when comparing participants on the day before symptoms developed, on the first day of the symptom, on the day following the first day of the symptom, or on all other days (Figure 3D-E).

**Table 4:** Number of participants reporting each symptom (N=297).

Symptom	Participants, n (%)^a^
Fever or chills	11 (3.7)
Fatigue or weakness	87 (29.3)
Body aches	47 (15.8)
Dry cough	32 (10.8)
Sneezing	52 (17.5)
Runny nose	43 (14.4)
Diarrhea	33 (11.1)
Sore throat	60 (20.2)
Headache	82 (27.6)
Shortness of breath	11 (3.7)
Loss of smell or taste	5 (1.7)
Itchy eyes	53 (17.8)
Other	26 (8.8)

^a^Percentages add to >100% because participants could report one or more symptoms.

**Table 5:** Heart rate variability parameters on the first day of reported symptoms compared to all other symptom-free days.

Parameter	Value on the first day of symptoms (milliseconds), mean (95% CI)	Value on all other days (milliseconds), mean (95% CI)	Difference (95% CI)	*P* value
MESOR^a^	46.01 (43.37 to 48.77)	43.48 (41.77 to 45.27)	2.53 (0.82 to 4.36)	*.01* ^b^
Amplitude	2.58 (0.26 to 5.00)	5.30 (4.95 to 5.66)	–2.73 (–5.16 to 0.31)	*.01*
Acrophase	–2.21 (–2.83 to –1.58)	–2.44 (–2.49 to –2.39)	0.24 (–0.38 to 0.88)	.44

^a^MESOR: midline statistic of rhythm.

^b^Italic text indicates statistical significance.

**Table 6:** Comparison of heart rate variability parameters based on symptom state and the time periods before and after the first day of reported symptoms.

Parameter and first symptom state			Value (milliseconds), mean (95% CI)		Second symptom state		Value (milliseconds), mean (95% CI)		Difference (95% CI)		*P* value
**MESOR^a^**											
	1 day after first symptom day	44.52 (42.05 to 46.94)		Asymptomatic		43.49 (41.74 to 45.21)		1.03 (–0.64 to 2.67)		.21	
	1 day before first symptom day	43.84 (41.41 to 46.15)		Asymptomatic		43.49 (41.74 to 45.21)		0.34 (–1.46 to 2.23)		.73	
	First day of symptom	44.87 (42.42 to 47.18)		Asymptomatic		45.49 (41.74 to 45.21)		1.37 (–0.24 to 3.04)		.11	
	1 day before first symptom day	43.84 (41.41 to 46.15)		1 day after first symptom day		44.52 (42.05 to 46.94)		–0.69 (–3.72 to 2.47)		.66	
	First day of symptom	44.87 (42.42 to 47.18)		1 day after first symptom day		44.52 (42.05 to 46.94)		0.34 (–1.46 to 2.23)		.73	
	First day of symptom	44.87 (42.42 to 47.18)		1 day before first symptom day		43.84 (41.41 to 46.15)		1.03 (–0.64 to 2.67)		.21	
**Amplitude**											
	1 day after first symptom day	5.47 (3.16 to 7.76)		Asymptomatic		5.32 (4.99 to 5.66)		0.15 (–2.21 to 2.37)		.91	
	1 day before first symptom day	2.92 (0.50 to 5.33)		Asymptomatic		5.32 (4.99 to 5.66)		–2.40 (–4.75 to –0.07)		.06	
	First day of symptom	3.07 (0.88 to 5.22)		Asymptomatic		5.32 (4.99 to 5.66)		–2.25 (–4.38 to –0.27)		*.04* ^b^	
	1 day before first symptom day	2.92 (0.50 to 5.33)		1 day after first symptom day		5.47 (3.16 to 7.76)		–2.55 (–6.64 to 1.65)		.25	
	First day of symptom	3.07 (0.88 to 5.22)		1 day after first symptom day		5.47 (3.16 to 7.76)		–2.40 (–4.75 to –0.06)		.06	
	First day of symptom	3.07 (0.88 to 5.22)		1 day before first symptom day		2.92 (0.50 to 5.33)		0.15 (–2.20 to 2.37)		.91	
**Acrophase**											
	1 day after first symptom day	–2.30 (–2.60 to –2.00)		Asymptomatic		–2.45 (–2.50 to –2.39)		0.14 (–0.15 to 0.44)		.33	
	1 day before first symptom day	–2.52 (–3.31 to –1.71)		Asymptomatic		–2.45 (–2.50 to –2.39)		–0.08 (–0.79 to 0.66)		.86	
	First day of symptom	–2.26 (–2.73 to –1.79)		Asymptomatic		–2.45 (–2.50 to –2.39)		0.19 (–0.24 to 0.63)		.36	
	1 day before first symptom day	–2.52 (–3.31 to –1.71)		1 day after first symptom day		–2.30 (–2.60 to –2.00)		–0.22 (–1.11 to 0.70)		.63	
	First day of symptom	–2.26 (–2.73 to –1.79)		1 day after first symptom day		–2.30 (–2.60 to –2.00)		0.04 (–0.36 to 0.46)		.86	
	First day of symptom	–2.26 (–2.73 to –1.79)		1 day before first symptom day		–2.52 (–3.31 to –1.71)		0.26 (–0.40 to 0.92)		.41	

^a^MESOR: midline statistic of rhythm.

^b^Italic text indicates statistical significance.

## Discussion

### Principal Results and Comparison with Prior Work

In this prospective study, longitudinally evaluated HRV metrics were found to be associated with a positive SARS-CoV-2 diagnosis and COVID-19 symptoms. Significant changes in these metrics were observed 7 days prior to the diagnosis of COVID-19. To the best of our knowledge, this is the first study to demonstrate that physiological metrics derived from a commonly worn wearable device (Apple Watch) can identify and predict SARS-CoV-2 infection prior to diagnosis with a SARS-CoV-2 nasal swab PCR test. These preliminary results identify a novel, easily measured physiological metric that may aid in the tracking and identification of SARS-CoV-2 infections.

Current means to control COVID-19 spread rely on case isolation and contact tracing, which have played major roles in the successful containment of prior infectious disease outbreaks [18-20]. However, due to the variable incubation period, high percentage of asymptomatic carriers, and infectivity during the presymptomatic period of COVID-19, containment of the disease has been challenging [21]. This has further limited the utility of systematic screening technologies reliant on vital sign assessment or self-reporting of symptoms [7]. Advances in digital health provide a unique opportunity to enhance disease containment. Wearable devices are commonly used and well accepted for health monitoring [9,22]. Commercially available devices are able to continually collect several physiological parameters. Unlike app-based platforms, wearable devices have the advantage of not requiring users to actively participate aside from regular use of the device. Prior to the COVID-19 pandemic, population-level data from the Fitbit wearable device demonstrated effectiveness of real-time geographic surveillance of influenza-like illnesses through the assessment of physiological parameters [23]. This concept was recently expanded during the COVID-19 pandemic by Quer and colleagues [10], who demonstrated that the combination of symptom-based data with resting heart rate and sleep data from wearable devices was superior to relying on symptom-based data alone to identify COVID-19 infections.

HRV has been shown to be altered during illnesses, with several small studies demonstrating changes in HRV associated with and predictive of the development of infection [24]. Ahmad and colleagues [16] followed 21 subjects undergoing bone marrow transplant, and they found a significant reduction in root mean square successive difference metrics prior to the clinical diagnosis of infection. Furthermore, wavelet HRV was noted to decrease by 25% on average 35 hours prior to a diagnosis of sepsis in 14 patients. In another study in 100 infants [15], significant HRV changes were noted 3-4 days preceding sepsis or systemic inflammatory response syndrome, with the largest increase being seen 24 hours prior to development. Building on these observations demonstrating that ANS changes accompany or precede infection, our team launched the Warrior Watch Study.

We demonstrated that significant changes in the circadian pattern of HRV, specifically the amplitude of SDNN, were associated with a positive COVID-19 diagnosis. Interestingly, when we compared these changes over the 7 days preceding the diagnosis of COVID-19, we continued to see significant alterations in amplitude when compared to individuals without COVID-19. This demonstrates the predictive ability of this metric to identify infection. Additionally, most participants diagnosed with COVID-19 in our cohort were asymptomatic. We demonstrated that there was no difference in changes in HRV metrics between participants with and without symptomatic COVID-19 infections. These findings support the utility of using wearable technology to identify COVID-19 infections, even in asymptomatic individuals. When we followed individuals 7-14 days after diagnosis with COVID-19, we found that the circadian HRV pattern began to normalize and was no longer statistically different from an uninfected pattern. As an exploratory analysis, we evaluated how HRV was impacted by symptoms associated with a COVID-19 diagnosis, as individuals may not be tested despite experiencing symptoms. We found significant changes in the amplitude of the circadian HRV pattern on the first day of symptoms, with a trend toward statistical significance on the days before and after symptoms were reported. Similarly, we found significant changes in HRV when we stratified subjects based on severe or nonsevere symptom severity. Taken together, these findings highlight the possible use of HRV collected via wearable devices to identify and predict COVID-19 infection.

### Limitations

There are several limitations to our study. First, the number of participants who were diagnosed with COVID-19 in our cohort was small, limiting our ability to determine how predictive HRV can be of infection. However, these preliminary findings support the further evaluation of HRV as a metric to identify and predict COVID-19 and warrant further study. An additional limitation is the sporadic collection of HRV by the Apple Watch. Although our statistical modeling was able to account for this, a denser dataset would enable expanded evaluation of the relationship between this metric and infections/symptoms. The Apple Watch also only provides HRV in one time domain (SDNN), limiting assessment of the relationship between other HRV parameters with COVID-19 outcomes. Additionally, we did not capture the times of day during which participants were awake or sleeping. Therefore, fluctuations in sleep patterns may have impacted some HRV readings and could not be controlled in the analysis. Finally, an additional limitation is that we relied on self-reported data in this study, precluding independent verification of COVID-19 diagnosis.

### Conclusions

In summary, we demonstrated a relationship between longitudinally collected HRV acquired from a commonly used wearable device and SARS-CoV-2 infection. These preliminary results support the further evaluation of HRV as a biomarker of SARS-CoV-2 infection by remote sensing. Although further study is needed, our findings may enable the identification of SARS-CoV-2 infection during the presymptomatic period, in asymptomatic carriers, and prior to diagnosis by a SARS-CoV-2 nasal swab PCR test. These findings warrant further evaluation of this approach to track and identify COVID-19 infections and possibly other types of infection.

## Appendix

Multimedia Appendix 1 Supplementary material.

## Abbreviations

ANS: autonomic nervous system
HRV: heart rate variability
IBI: interbeat interval
ISMMS: Icahn School of Medicine at Mount Sinai
MESOR: midline statistic of rhythm
PCR: polymerase chain reaction
REWL: reweighted least squares
SDNN: standard deviation of the interbeat interval of normal sinus beats
