# Supplementary material for: Use of Physiological Data From a Wearable Device to Identify SARS-CoV-2 Infection and Symptoms and Predict COVID-19 Diagnosis: Observational Study
Source: J Med Internet Res. 2021 Feb 22;23(2):e26107. doi: 10.2196/26107 (PMC7901594; doi:10.2196/26107)

Table S1. Infection related survey questions.

| Survey Question | Survey Responses |
| --- | --- |
| Have you had any of the following symptoms in the past 24 hours? Check all boxes that apply: | Fever or high temperature, chills, tired or weak, body aches, dry cough, sneezing, runny nose, diarrhea, sore throat, headache, shortness of breath, loss of smell or taste, itchy eyes, none, other [insert], None of these |
| On a scale of 1 - 10, how bad do your symptoms feel today, compared to colds or flu that you have had in the past? | 1 [Extremely mild symptoms], 2, 3, 4, 5, 6, 7, 8, 9, 10 [The worst symptoms that you have ever had] |
| If you have been tested for the Coronavirus by nasal PCR since starting this study, what was the result of your test: | Positive / Negative / I have not been tested for Coronavirus/Prefer not to say |
| If you were tested for the Coronavirus by nasal PCR since starting the study, what is the date you were tested? | Date: __________ |
| If you have been tested for the Coronavirus by blood antibody since starting this study, what was the result of your test: | Positive / Negative / I have not been tested for Coronavirus/Prefer not to say |
| If you were tested for the Coronavirus by blood antibody since starting the study, what is the date you were tested? | Date: __________ |

Table S2. Comparison of longitudinal heart rate variability metrics between participants who never received a COVID-19 diagnosis versus those with a positive COVID-19 nasal PCR at enrollment.

| Parameter | Parameter Mean, ms (95% CI) Never COVID-19 Positive | Parameter Mean, ms (95% CI) COVID-19 Positive | Difference in Parameter Mean, ms (95% CI)  No positive nasal PCR vs Positive Nasal PCR at Enrollment | Standard Error | *P*-value |
| --- | --- | --- | --- | --- | --- |
| MESOR | 43.71 (41.83-45.54) | 47.63 (41.74-54.08) | 3.92 (-2.39- 10.17) | 3.20 | 0.22 |
| Amplitude | 5.33 (4.96-5.70) | 5.54 (4.30- 6.75) | 0.21 (-0.93- 1.39) | 0.59 | 0.73 |
| Acrophase | -2.45 (-2.50- -2.39) | -2.36 (-2.52- -2.19) | 0.09 (-0.08- 0.25) | 0.09 | 0.27 |

Table S3. Comparison of heart rate variability metrics between participants with a positive COVID-19 nasal PCR during follow-up versus those with a negative COVID-19 nasal PCR during follow-up.

| Parameter | Parameter Mean, ms (95% CI) COVID-19 Negative | Parameter Mean, ms (95% CI) COVID-19 Positive | Difference in Parameter Mean, ms (95% CI)  Negative Nasal PCR vs Positive Nasal PCR | Standard Error | *P*-value |
| --- | --- | --- | --- | --- | --- |
| MESOR | 43.44 (40.68-46.16) | 39.47 (34.24-44.76) | -3.97 (-8.77- 0.79) | 2.44 | 0.12 |
| Amplitude | 7.66 (6.87-8.42) | 2.62 (-1.08- 5.89) | -5.04 (-8.83- 1.71) | 1.82 | **0.004** |
| Acrophase | -2.55 (-2.64- -2.46) | -3.72 (-5.66- -1.99) | -1.17 (-2.93- 0.53) | 0.88 | 0.16 |

Figure S1. Locally estimated scatterplot smoothing (loess) curve showing a daily circadian pattern on HRV measures. Such pattern can be represented by the COSINOR model using 3 parameters: the rhythm-adjusted mean (MESOR), half the extent of variation within a day (Amplitude) and the time of overall high values recurring in each day (acrophase). Red and green dots represent hypothetical sampling times though the day from two subjects that have the same daily curve, showing that features like maximum, range, or CV will be easily biased by the sampling time.


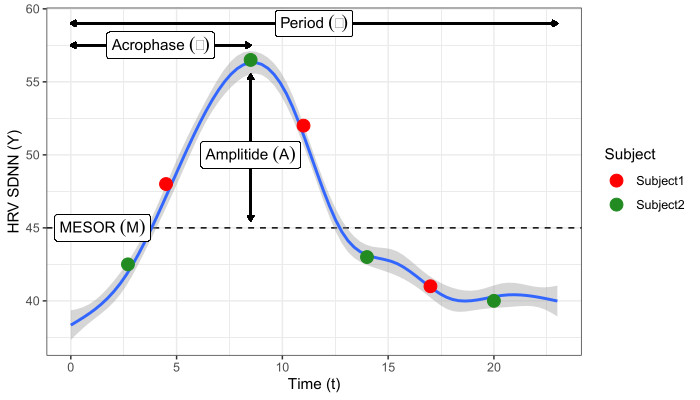

Supplement: Multimedia Appendix 1 [file jmir_v23i2e26107_app1.docx]
